# The Impact of Assisted Reproduction Technology on Breast Cancer Risk for Women With Infertility: Current Insights and Future Perspectives

**DOI:** 10.7759/cureus.116210

**Published:** 2026-09-14

**Authors:** Kanelina Bimpa, Emmanouil M Xydias, Dimitrios Varvaras, Dimitrios N Koronarchis, Elias Tsakos

**Affiliations:** 1 Department of Breast Health, BreastCare Outpatient Clinic, Thessaloniki, GRC; 2 Department of Breast Health, EmbryoClinic IVF, Thessaloniki, GRC; 3 Department of Obstetrics and Gynecology, EmbryoClinic IVF, Thessaloniki, GRC; 4 Department of Breast Surgery, Breast Health Clinic Pio XI, Rome, ITA; 5 3rd Breast Clinic, Metropolitan General, Athens, GRC

**Keywords:** assisted reproduction technology, breast cancer, clomiphene citrate, controlled ovarian stimulation, fertility preserving, infertility, in vitro fertilization (ivf), letrozole, ovulation induction

## Abstract

Breast cancer is the most frequently diagnosed malignancy among women worldwide. Because assisted reproduction technology (ART) commonly involves controlled ovarian stimulation (COS) and temporary increases in circulating estradiol and progesterone, its possible effect on long-term breast cancer risk remains an important clinical question. This narrative review, supported by a structured literature search of MEDLINE/PubMed, Scopus, and Web of Science from January 2003 to March 2026, summarizes 31 primary observational studies with a cumulative reported sample exceeding 7.1 million participants, with follow-up extending beyond three decades in some cohorts. Most studies found no significant association between ART and breast cancer incidence. Signals of increased risk were reported in selected subgroups, including women undergoing multiple stimulation cycles, women starting treatment at younger or older ages, and some women with prolonged clomiphene citrate (CC) exposure. The review also considers evidence concerning letrozole, postpartum breast cancer risk, tumor subtype, and fertility preservation in women with breast cancer. Overall, the available evidence does not demonstrate a meaningful increase in breast cancer risk attributable to ART itself. However, substantial heterogeneity, the predominance of older treatment protocols, and the lack of long-term incidence data for contemporary letrozole-based treatment limit definitive conclusions. Prospective studies using current ART protocols and long-term, subtype-specific follow-up are needed.

## Introduction and background

Breast cancer is the most commonly diagnosed cancer in women, accounting for more than 2.3 million new diagnoses globally in 2022 alone [1]. At the same time, outcomes in this disease are comparatively favorable: owing to widespread screening, heightened public awareness, and continuous therapeutic progress, the five-year relative survival rate across all stages combined now approaches 91% in contemporary registry data [2]. The combination of high incidence and high survivorship means that an ever-larger population of women of reproductive age either carries a meaningful lifetime probability of developing breast cancer or has already survived it. Any medical intervention delivered to this population - particularly one acting on the hormonal milieu - therefore deserves careful scrutiny with respect to breast cancer risk.

The recognized determinants of breast cancer span several domains. Heritable factors include a positive family history and pathogenic variants in the breast cancer susceptibility genes 1 and 2 (BRCA1 and BRCA2); reproductive and endocrine factors include early menarche, late menopause, nulliparity, advanced maternal age at first birth, and the use of exogenous hormones; lifestyle and environmental contributors include alcohol consumption, tobacco smoking, obesity, and prior thoracic radiotherapy [3]. A unifying thread among most of these factors - and the one most relevant to the hormone receptor-positive subtype that constitutes the majority of cases - is the duration and intensity of estrogen exposure over a woman’s lifetime [3].

In parallel, infertility now affects a substantial and growing proportion of couples worldwide, and the use of assisted reproduction technology (ART) - principally in vitro fertilization (IVF) and intracytoplasmic sperm injection (ICSI), together with simpler ovulation-induction strategies - has expanded accordingly, such that many millions of children worldwide have now been conceived through these techniques and ART accounts for a meaningful proportion of all births in several countries. The cornerstone of most ART protocols is controlled ovarian stimulation (COS), in which agents such as clomiphene citrate (CC), human menopausal gonadotropin (hMG), recombinant gonadotropins (RG), human chorionic gonadotropin (hCG), and gonadotropin-releasing hormone (GnRH) analogues are administered to recruit multiple ovarian follicles. Multifollicular development transiently raises serum estradiol to concentrations far in excess of those observed during a spontaneous ovulatory cycle, and luteal support frequently adds exogenous progesterone to this hormonal load. Prescribing practice itself has evolved considerably over the period covered by the epidemiological literature: letrozole, an aromatase inhibitor that induces ovulation while transiently suppressing rather than elevating estradiol, has progressively displaced CC as the first-line agent for anovulatory infertility [4,5], a shift whose implications for long-term safety research are examined in this review.

Because breast cancer is hormonally responsive and ART can produce temporary increases in reproductive hormone concentrations, the possible relationship between fertility treatment and subsequent breast cancer risk warrants careful evaluation. Interpretation is complicated by the characteristics of women receiving fertility treatment: they are often older and nulliparous, both of which can independently influence breast cancer risk. Separating the effect of treatment from the effect of infertility and reproductive history is therefore difficult, particularly because breast cancer may develop many years after exposure.

This narrative review evaluates evidence published between January 2003 and March 2026 concerning breast cancer incidence after ART in women with infertility. It also examines subgroups in which different risk patterns have been reported, including women exposed to multiple treatment cycles and those receiving CC. Additional sections address letrozole-based ovulation induction, postpartum breast cancer risk, tumor molecular subtype, and fertility preservation in women with breast cancer. The review concludes by translating the available evidence into a practical framework for counseling and identifying priorities for future research.

## Review

Methods

Study Design and Research Question

This work was designed as a narrative review underpinned by a structured and reproducible literature search, rather than a formal systematic review with quantitative pooling; the rationale for this choice is set out under “Data synthesis” below. The primary research question was framed according to the Population, Intervention, Comparator, Outcome (PICO) schema as follows: Population: women aged 18 years or older with a diagnosis of infertility; Intervention: exposure to any modality of ART, including IVF, ICSI, ovulation induction, and COS with any associated pharmacological agents; Comparators: women with infertility who did not receive ART, or the general female population; Outcome: incident breast cancer, expressed as an odds ratio (OR), risk ratio (RR), hazard ratio (HR), or standardized incidence ratio (SIR), each accompanied by a 95% confidence interval (CI).

Eligibility Criteria

Studies were considered eligible for inclusion if they satisfied all of the following criteria: (i) primary observational design, namely prospective or retrospective cohort, case-control, or cross-sectional; (ii) enrollment of adult women (18 years or older) with documented infertility; (iii) exposure to any ART modality or to ovulation-induction pharmacotherapy, including CC, hMG, RG, GnRH analogues, hCG, or progesterone; (iv) reporting of breast cancer incidence in the exposed group relative to an internal or external comparator, with an extractable effect estimate; (v) inclusion of at least five breast cancer cases; and (vi) publication between 01 January 2003 and 01 March 2026.

The following were grounds for exclusion: secondary literature, such as narrative reviews, systematic reviews, and meta-analyses, which were retrieved and consulted for context and for the discussion but were not eligible for data extraction; case reports and case series describing fewer than five cases; editorials, commentaries, and letters to the editor; conference abstracts without an accompanying full-text publication; animal or purely in vitro investigations; and studies from which no quantitative risk estimate for breast cancer could be derived.

Information Sources and Search Strategy

MEDLINE/PubMed, Scopus, and Web of Science were interrogated from 01 January 2003 through 01 March 2026. The search string combined Medical Subject Headings (MeSH) with free-text keywords in the following Boolean structure: “assisted reproductive techniques” [MeSH Terms] OR “in vitro fertilization” OR “in vitro fertilisation” OR “intracytoplasmic sperm injection” OR “IVF” OR “ICSI”, combined with “breast neoplasms” [MeSH Terms] OR “breast” AND (“cancer” OR “carcinoma*” OR “neoplasm” OR “tumour” OR “tumor” OR “malignancy”). No automated filters or language restrictions were applied at the search stage. The reference lists of all eligible articles and of previously published reviews on the topic were additionally hand-searched to identify records not captured by the electronic strategy. For the contextual sections of the discussion concerning letrozole, postpartum breast cancer risk, tumor subtype, and fertility preservation in women with breast cancer, targeted supplementary searches of the same databases were performed; literature identified in this way was used for interpretation and discussion only and was not subject to data extraction.

Study Selection

All retrieved records were exported to a common library and de-duplicated. Titles and abstracts were screened for relevance to the research question by three authors working independently, and any record judged potentially eligible by at least one reviewer proceeded to full-text assessment. Full texts were then evaluated against the eligibility criteria by two authors; disagreements at either stage were resolved through discussion, with arbitration by the senior author where consensus could not initially be reached.

Data Extraction

Data were extracted in duplicate onto a piloted, standardized collection form. The following parameters were recorded for each study: study characteristics (first author, year of publication, country of origin, design, and recruitment or observation window); population characteristics (total sample size, numbers of exposed cases and comparators, mean or median duration of follow-up, and the confounding factors adjusted for in the analysis, such as age, parity, body mass index (BMI), smoking, alcohol intake, personal and family history of breast cancer, use of oral contraceptives or hormone replacement therapy, age at menarche, menopausal status, race, and socioeconomic status); exposure characteristics (type of ART, medications administered, and, where reported, number of treatment cycles); and outcome data (adjusted and unadjusted effect estimates with their 95% CI).

Data Synthesis and Statistical Considerations

The included studies differed substantially in design, comparator population (untreated infertile women versus the general population), exposure definition, duration of follow-up, and the statistical measure used to express risk (OR, RR, HR, or SIR). Because these measures are not directly interchangeable and the underlying populations were heterogeneous, formal quantitative pooling was judged methodologically inappropriate for the purposes of this review, and a qualitative narrative synthesis was performed instead; readers seeking pooled estimates are directed to the meta-analyses discussed later in this article. Extracted data were tabulated to allow side-by-side comparison of study characteristics (Table 1) and of principal findings and conclusions (Table 2). Where a study reported both crude and adjusted estimates, the adjusted figure was preferred. An association was described as statistically significant when the reported 95% CI excluded unity.

**Table 1:** Summary of the main characteristics of the included studies. Abbreviations: ART = assisted reproduction technology; BMI = body mass index; CC = clomiphene citrate; GnRH = gonadotropin-releasing hormone; hCG = human chorionic gonadotropin; hMG = human menopausal gonadotropin; IVF = in vitro fertilization; MAR = medically assisted reproduction; NR = not reported; RG = recombinant gonadotropins; UK = United Kingdom; USA = United States of America

Authors	Year	Design	Country	Duration	Sample size	Follow-up (mean)	Confounding factors adjusted for	Treatment type
Lerner-Geva et al. [6]	2003	Cohort	Israel	1984-1992	Total: 1,082	6.5 years	Infertility	NR
Burkman et al. [7]	2003	Case-control	USA	1994-1998	Total: 9,257; Cases: 4,575; Controls: 4,682	NR	Age, race	CC, hMG
Gauthier et al. [8]	2004	Cohort	France	1990-2000	Total: 92,555; Infertility treatment: 6,602; No fertility treatment: 85,953	9.7 years	BMI, smoking, personal and family history of breast cancer, age at menarche, menopause, infertility	CC, RG
Brinton et al. [9]	2004	Cohort	USA	1965-1988	Total: 12,193	18.8 years	Age, infertility, family history of breast cancer	CC, RG
Lerner-Geva et al. [10]	2006	Cohort	Israel	1964-1984	Total: 5,788	13.1 years	Age, infertility, use of contraceptives	CC, hMG
Kristiansson et al. [11]	2007	Cohort	Sweden	1981-2001	Total: 647,704; IVF: 8,716; Non-IVF: 638,988	IVF: 6.2 years; Non-IVF: 7.8 years	Age, Infertility	NR
Jensen et al. [12]	2007	Cohort	Denmark	1963-1998	Total: 54,362	8.8 years	Infertility	RG, hCG, hMG, GnRH
Pappo et al. [13]	2008	Cohort	Israel	1986-2003	Total: 3,375	8.1 years	Age, infertility, family history	NR
Silva Idos et al. [14]	2009	Cohort	UK	1963-1999	Total: 7,355	21.4 years	Age, infertility	CC, RG
Calderon-Margalit et al. [15]	2009	Cohort	Israel	1974-2004	Total: 15,030; Ovulation induction: 567; No treatment: 14,463	29.0 years	Age, infertility, socioeconomic status, geographic origin	CC
Orgéas et al. [16]	2009	Cohort	Sweden	1961-1976	Total: 1,135	30.9 years	Age, infertility	CC, RG
Källén et al. [17]	2011	Cohort	Sweden	1982-2006	Total: 1,394,061 IVF: 24,058; Non-IVF: 1,370,003	NR	Age, infertility, smoking	NR
Lerner-Geva et al. [18]	2012	Cohort	Israel	1964-1974	Total: 2,431	33.8 years	Age	CC, hMG
Stewart et al. [19]	2012	Cohort	Australia	1983-2002	Total: 21,025; IVF: 7,381; Non-IVF: 13,644	16.3 years	Age, infertility, race	NR
Yli-Kuha et al. [20]	2012	Cohort	Finland	1996-2004	Total: 18,350; Infertility-treated: 9,175; Controls: 9,175	7.8 years	Socioeconomic status, marriage	NR
Fei et al. [21]	2012	Case-control	Worldwide	2008-2010	Total: 3,091; Cases: 1,422; Controls: 1,669	NR	Age, infertility, smoking, alcohol	CC, RG
Brinton et al. [22]	2013	Cohort	Israel	1994-2011	Total: 87,403	8.1 years	Age, infertility, smoking, alcohol	GnRH, CC, progesterone
Brinton et al. [23]	2014	Cohort	USA	1965-1988	Total: 9,892	30.0 years	Age, infertility, family history	CC, RG
Reigstad et al. [24]	2015	Cohort	Norway	1984-2010	Total: 808,834; ART: 16,626; Non-ART: 792,208	16.0 years	Age, infertility, residence	NR
Luke et al. [25]	2015	Cohort	USA	2004-2009	Total: 113,226;	4.8 years	Age, infertility, medication dosage	CC, RG
Kessous et al. [26]	2016	Cohort	Israel	1988-2013	Total: 106,031; Fertility treatment: 4,363; No fertility treatment: 101,668	11.6 years	Age, infertility, BMI	NR
van den Belt-Dusebout et al. [27]	2016	Cohort	Netherlands	1980-1995	Total: 25,108; IVF: 19,158; Non-IVF: 5,950	21.1 years	Age, infertility, family history	IVF
Reigstad et al. [28]	2017	Cohort	Norway	1960-1996	Total: 1,353,724; Fertility treatment: 56,194; No fertility treatment: 1,297,530	11.0 years	Age, infertility	RG, CC, other
Lundberg et al. [29]	2017	Cohort	Sweden	1982-2012	Total: 1,340,211	ART birth: 9.6 years; No ART birth: 14.6 years	Age, parity, family history	CC, RG
Derks-Smeets et al. [30]	2018	Cohort	Netherlands	2010-2013	Total: 2,514; IVF-exposed: 76; IVF-unexposed: 2,434; IVF status missing: 4	NR	Age	NR
Williams et al. [31]	2018	Cohort	UK	1991-2002	Total: 255,786	8.8 years	Age, infertility, personal history	NR
Taheripanah et al. [32]	2018	Case-control	Iran	2011-2013	Total: 1,856; Cases: 928; Controls: 928	NR	Age, infertility, family history, contraceptives and hormonal treatment	CC
Tsafrir et al. [33]	2020	Cohort	Israel	1994-2002	Total: 501	16.7 years	Age, infertility	NR
Perri et al. [34]	2021	Cohort	Israel	1995-2019	Total: 1,824; Fertility treatment: 332; No fertility treatment: 1,492	NR	Age, infertility, contraceptives and hormonal treatment	CC, RG
Vassard et al. [35]	2021	Cohort	Denmark	1994-2016	Total: 641,339 ART: 61,579; Non-ART: 579,760	ART: 9.69 years; Non-ART: 9.28 years	Age, infertility, family history	NR
Machtinger et al. [36]	2022	Cohort	Israel	1994-2019	Total: 97,098 ART: 32,366; MAR: 32,366; Controls: 32,366	9.1 years	Age, infertility, smoking	hMG, RG

**Table 2:** Summary of the main outcomes and conclusions of the included studies. Abbreviations: ART = assisted reproduction technology; BRCA1/2 = breast cancer susceptibility genes 1 and 2; CC = clomiphene citrate; CI = confidence interval; HR = hazard ratio; IVF = in vitro fertilization; OR = odds ratio; RR = risk ratio; SIR = standardized incidence ratio

Authors	Year	Main endpoint(s)	Results	Study conclusions
Lerner-Geva et al. [6]	2003	Breast cancer risk	Breast cancer incidence after ART was in line with expected estimates (SIR = 1.02, 95% CI 0.33-2.39).	No significant association between ART and breast cancer risk.
Burkman et al. [7]	2003	Breast cancer risk	No association with breast cancer risk overall (OR = 0.9, 95% CI 0.8-1.2); at least six cycles carried significant risk in women with infertility (OR = 3.8, 95% CI 1.2-11.8).	No significant association overall, except with prolonged use (more than six cycles).
Gauthier et al. [8]	2004	Breast cancer risk	No association with breast cancer risk (RR = 0.95, 95% CI 0.82-1.11).	No significant association between ART and breast cancer risk.
Brinton et al. [9]	2004	Breast cancer risk	Infertility itself was linked to higher risk (SIR = 1.29, 95% CI 1.1-1.4); no significant effect of individual ART treatments; significant association of CC after extended observation (RR = 1.60, 95% CI 1.0-2.5).	No overall risk increase; significant increase with CC over long follow-up.
Lerner-Geva et al. [10]	2006	Breast cancer risk	Risk after ART comparable to the general population (SIR = 1.1, 95% CI 0.9-1.4); significantly higher with CC use (SIR = 1.4, 95% CI 1.0-1.8).	No association for ART overall; increased risk with CC use.
Kristiansson et al. [11]	2007	Breast cancer risk	No association of ART with breast cancer risk (RR = 0.94-1.28); progesterone use was linked to an increase (RR = 3.36, 95% CI 1.3-8.6).	No significant association, with the exception of progesterone.
Jensen et al. [12]	2007	Breast cancer in situ risk	No significant association with ART (adjusted RR = 0.93, 95% CI 0.58-1.43).	No significant association between ART and breast cancer risk.
Pappo et al. [13]	2008	Breast cancer risk	Non-significant elevation overall versus the non-ART cohort (SIR = 1.4, 95% CI 0.98-1.96); four or more IVF cycles carried higher risk (SIR = 2.0, 95% CI 1.15-3.27).	Increased risk in women undergoing more than four IVF cycles.
Silva Idos et al. [14]	2009	Breast cancer risk	Non-significant elevation versus the non-ART cohort (SIR = 1.13, 95% CI 0.97-1.30).	No significant association between ART and breast cancer risk.
Calderon-Margalit et al. [15]	2009	Breast cancer risk	Ovulation induction showed a non-significant risk increase (multivariate HR = 1.42, 95% CI 0.99-2.05).	No significant association between ART and breast cancer risk.
Orgéas et al. [16]	2009	Breast cancer risk	No significant impact of ART on breast cancer risk (SIR = 1.01, 95% CI 0.77-1.31).	No significant association between ART and breast cancer risk.
Källén et al. [17]	2011	Breast cancer risk	ART was linked to lower cancer risk than in other non-nulliparous women (OR = 0.76, 95% CI 0.62-0.94).	ART associated with decreased breast cancer risk.
Lerner-Geva et al. [18]	2012	Breast cancer risk	Non-significant elevation versus the non-ART cohort (SIR = 1.1, 95% CI 0.98-1.36).	No significant association between ART and breast cancer risk.
Stewart et al. [19]	2012	Breast cancer risk	ART not linked to elevated risk overall (HR = 1.10, 95% CI 0.88-1.36); risk elevated in women aged 24 years or younger at the start of ART (HR = 1.59, 95% CI 1.05-2.42).	No overall association; increased risk in women starting treatment at a young age.
Yli-Kuha et al. [20]	2012	Breast cancer risk	ART showed no measurable effect on breast cancer risk (OR = 0.93, 95% CI 0.62-1.40).	No significant association between ART and breast cancer.
Fei et al. [21]	2012	Breast cancer risk	No association of ART with breast cancer risk (OR = 0.82, 95% CI 0.63-1.08).	No significant association between ART and breast cancer risk.
Brinton et al. [22]	2013	Breast cancer risk	No association of ART with breast cancer risk (HR = 0.87, 95% CI 0.71-1.06).	No association between breast cancer risk and ART.
Brinton et al. [23]	2014	Breast cancer risk	No risk increase with CC (HR = 1.05, 95% CI 0.90-1.22); increase with gonadotropins among nulligravid patients (HR = 1.98, 95% CI 1.04-3.60).	Ovulation induction not associated with increased risk; gonadotropin use showed a modest association.
Reigstad et al. [24]	2015	Breast cancer risk	ART linked to elevated breast cancer risk (HR = 1.20, 95% CI 1.01-1.42), more pronounced with more extensive observation (HR = 1.35, 95% CI 1.07-1.71).	Increased breast cancer risk in women undergoing ART.
Luke et al. [25]	2015	Breast cancer risk	Decreased risk in the ART group versus the non-ART cohort (SIR = 0.83, 95% CI 0.75-0.91).	Lower breast cancer risk after ART.
Kessous et al. [26]	2016	Breast cancer risk	Incidence was comparable between the ART group and controls (n = 1,149, 0.4% versus n = 3,214, 0.5%); no significant risk increase (p = 0.926).	No significant association between ART and breast cancer risk.
van den Belt-Dusebout et al. [27]	2016	Breast cancer risk	Risk after ART comparable to the non-ART cohort (SIR = 1.01, 95% CI 0.93-1.09); no increase with longer time since treatment (SIR = 0.92, 95% CI 0.73-1.15).	No significant association, irrespective of time elapsed since treatment.
Reigstad et al. [28]	2017	Breast cancer risk	No association of ART overall (HR = 1.00, 95% CI 0.81-1.22); no association with CC use overall (HR = 1.12, 95% CI 0.93–1.35), but an increased risk among parous women exposed to CC (HR = 1.26, 95% CI 1.03–1.54).	No overall association with ART or CC use; increased risk among parous women exposed to CC.
Lundberg et al. [29]	2017	Breast cancer risk	Risk was lower in women giving birth after ART than after spontaneous conception (HR = 0.84, 95% CI 0.74-0.95).	Inverse association between ART and breast cancer risk.
Derks-Smeets et al. [30]	2018	Breast cancer risk in BRCA1/2 carriers	No association of ART with breast cancer risk (HR = 0.79, 95% CI 0.46-1.36), nor of infertility itself (HR = 0.73, 95% CI 0.39-1.37).	No association between ovarian stimulation and breast cancer risk in BRCA1/2 mutation carriers.
Williams et al. [31]	2018	Breast cancer risk (different cancer types)	No association of ART with invasive breast cancer (SIR = 0.98, 95% CI 0.94-1.01); increased in situ breast carcinoma (SIR = 1.15, 95% CI 1.02-1.29), dependent on cycle number.	No overall association; an association with in situ breast carcinoma only.
Taheripanah et al. [32]	2018	Breast cancer risk	Ovulation induction showed no association with breast cancer risk (OR = 1.13, 95% CI 0.7-1.85).	No significant association of ovulation induction with breast cancer risk.
Tsafrir et al. [33]	2020	Invasive breast cancer risk in older women	Invasive breast carcinoma occurred in 22 women versus 19.84 expected (SIR = 1.11, 95% CI 0.69-1.68).	No additional risk in women aged 40 years or older undergoing IVF.
Perri et al. [34]	2021	Breast cancer risk	No significant association with breast cancer risk for CC (HR = 0.77, 95% CI 0.49–1.19), gonadotropins (HR = 0.54, 95% CI 0.28–1.01), IVF (HR = 0.65, 95% CI 0.39–1.08), or combined treatments (HR = 1.23, 95% CI 0.49–3.06).	No significant association between fertility treatment and breast cancer risk among BRCA1/2 mutation carriers.
Vassard et al. [35]	2021	Breast cancer risk	Slightly elevated risk in the ART cohort (HR = 1.14, 95% CI 1.12-1.16); age-dependent, particularly at 40 years or older at treatment start (HR = 1.37, 95% CI 1.29-1.45).	Small risk increase after ART, greater with advancing age, especially after 40 years.
Machtinger et al. [36]	2022	Breast cancer incidence and BRCA1/2 positivity prevalence	Breast carcinoma per 10,000 person-years: ART 11.9 (95% CI 10.7-13.3) versus non-ART 10.7 (95% CI 9.6-12.0); comparable risk (HR = 1.10, 95% CI 0.94-1.28); BRCA1/2 positivity prevalence similar.	Breast cancer risk was similar between ART patients and the general population.

Results

Study Selection and Characteristics

Thirty-one primary studies satisfied the eligibility criteria and were incorporated into the review [6-36]. These comprised 28 cohort studies and three case-control studies [7,21,32], published between 2003 and 2022, with observation windows collectively spanning 1961 to 2019. The cumulative reported sample across 31 included studies amounted to 7,134,141 participants, and mean follow-up ranged from 6.2 to 33.8 years across studies. However, this figure should not be interpreted as representing unique individuals, as some publications included overlapping populations, extended follow-up of previously reported cohorts, or analyses derived from the same national registries. The investigations originated from Israel, the United States of America (USA), France, Denmark, Sweden, Norway, Finland, the United Kingdom (UK), the Netherlands, Australia, Iran, and one multinational collaboration. Adjustment for potential confounders varied but most frequently encompassed age and underlying fertility status, with individual studies additionally accounting for race, smoking, alcohol intake, BMI, personal and family history of breast cancer, hormonal contraception or replacement therapy, age at menarche, menopausal status, parity, and socioeconomic indicators. The pharmacological exposures examined most often included CC, hMG, RG, GnRH analogues, hCG, and progesterone, while a considerable number of registry-based studies did not specify the regimen administered; notably, no eligible study reported long-term breast cancer incidence after letrozole-based ovulation induction in women without pre-existing malignancy. The principal characteristics of the included studies are presented in Table 1.

Overall Association Between ART and Breast Cancer Risk

Sixteen studies - the majority of the evidence base - detected no statistically significant relationship between exposure to ART and subsequent breast cancer incidence [6,8,12,14-16,18,20-22,26,27,30,32,33,36]. Three further studies reported point estimates below unity, compatible with a protective association among treated women, particularly those who achieved a live birth [17,25,29]. Only three studies concluded that ART exposure was independently associated with a measurable excess of breast cancer risk [23,24,35]: Brinton et al. observed an elevated hazard confined to nulligravid recipients of gonadotropins (HR = 1.98, 95% CI 1.04-3.60) [23]; Reigstad et al. reported a modest overall increase among parous Norwegian women (HR = 1.20, 95% CI 1.01-1.42) [24]; and Vassard et al. described a small excess in a Danish national cohort (HR = 1.14, 95% CI 1.12-1.16) that was amplified in women commencing treatment at 40 years of age or older (HR = 1.37, 95% CI 1.29-1.45) [35].

Subgroup Observations

Several studies that found no overall association nonetheless identified subgroups with a differential risk profile. Two investigations linked a high cumulative number of stimulation cycles to elevated risk, namely more than six cycles in the case-control analysis of Burkman et al. (OR = 3.8, 95% CI 1.2-11.8) [7] and four or more IVF cycles in the Israeli cohort of Pappo et al. (SIR = 2.0, 95% CI 1.15-3.27) [13]. Three studies observed an excess of breast cancer specifically among recipients of CC, particularly in relation to parity and extended follow-up [9,10,28]. Isolated subgroup signals were additionally described for progesterone supplementation (RR = 3.36, 95% CI 1.3-8.6) [11], for women younger than 24 years at treatment initiation (HR = 1.59, 95% CI 1.05-2.42) [19], and for carcinoma in situ of the breast (SIR = 1.15, 95% CI 1.02-1.29) [31]. The principal findings and conclusions of each included study are summarized in Table 2.

Discussion

Principal Findings

Across the 31 included studies, with a cumulative reported sample exceeding 7.1 million participants, most studies found no significant association between ART and breast cancer incidence. Three studies reported an increased risk and three reported a lower risk, although these findings were generally limited to particular subgroups. The following sections consider the main subgroup findings and the additional evidence relevant to their interpretation.

Number of Stimulation Cycles and Cumulative Exposure

Two studies reported higher breast cancer risk after multiple stimulation cycles. Burkman et al. found an increased risk after more than six cycles [7], while Pappo et al. reported a higher SIR after four or more IVF cycles [13]. These findings were not confirmed in the large Dutch OMEGA cohort, which found no association between either cycle number or time since treatment and subsequent breast cancer incidence [27]. Meta-analyses have also found no clear increase among women undergoing multiple IVF cycles [37]. The earlier findings may therefore reflect residual confounding, since women requiring repeated treatment may differ in age, ovarian reserve, and severity or duration of infertility.

Clomiphene Citrate

CC warrants separate consideration because it is a selective estrogen receptor modulator and was widely used for ovulation induction before the more recent shift toward letrozole. Three included studies reported higher breast cancer risk among CC users, particularly in relation to parity and longer follow-up [9,10,28]. Other studies did not confirm this association [23,32], and meta-analyses have not demonstrated a statistically significant increase in breast cancer incidence with CC [37,38]. Differences between studies may partly reflect confounding by indication, as CC was historically used in women with specific infertility characteristics and was sometimes administered over repeated courses. Continued observation of long-term outcomes remains appropriate, particularly in women with substantial historical exposure.

Letrozole and Aromatase Inhibition: An Evidence Gap of Growing Importance

Long-term breast cancer incidence data for letrozole in women without previous malignancy are limited. This is an important evidence gap because letrozole has become a first-line treatment for ovulation induction in many women with anovulatory infertility. The randomized trial by Legro et al. showed higher live birth rates with letrozole than with CC in women with polycystic ovary syndrome [4], and subsequent evidence has supported its use [5]. From a hormonal perspective, letrozole differs from CC because aromatase inhibition reduces estrogen production during treatment rather than producing the estrogenic exposure associated with stimulation. Available long-term safety data in reproductive medicine largely come from women with breast cancer undergoing fertility preservation, where letrozole-based stimulation has not been associated with an excess of recurrence [39,40]. These findings are reassuring but cannot replace long-term incidence data in women without previous cancer. Registry-based follow-up of women receiving letrozole for ovulation induction should therefore be a research priority.

Other Regimens and Demographic Subgroups

Isolated subgroup findings scattered across the literature resist firm interpretation but deserve acknowledgment. Kristiansson et al. linked progesterone supplementation to a substantially elevated relative risk, albeit with a wide confidence interval reflecting few exposed cases [11]. Brinton et al. observed an excess risk with gonadotropins that was confined to nulligravid women, raising the possibility of an interaction between treatment and the well-established protective effect of pregnancy [23]. Age at treatment initiation has produced signals at both extremes: Stewart et al. reported elevated risk in women commencing ART at 24 years of age or younger [19], whereas Vassard et al. found the greatest excess in women starting at 40 years or older [35], and Tsafrir et al., focusing specifically on patients treated at 40 years and beyond, found no additional risk at all [33]. Finally, Williams et al. detected an association restricted to carcinoma in situ, proportional to the number of cycles received [31]; because in situ lesions are disproportionately identified through imaging, this finding may partly reflect surveillance bias, as women who have undergone fertility treatment tend to interact more intensively with healthcare services and may therefore undergo earlier and more frequent breast assessment.

Pathophysiological Considerations

The biological rationale for concern is well established. COS increases endogenous estrogen and progesterone production, and estradiol concentrations during stimulated cycles can be substantially higher than during a spontaneous ovulatory cycle [19,41]. Estrogen can promote breast epithelial proliferation through receptor-mediated pathways and may also contribute to carcinogenesis through genotoxic mechanisms [42]. Progesterone can increase the progenitor cell compartment and promote DNA replication in normal breast tissue [43]. Estrogen metabolites and reactive oxygen species may provide additional mechanisms relevant to breast carcinogenesis [44,45]. However, the epidemiological findings remain largely null. ART-related hormonal exposure is intense but short, occurring over days during each treatment cycle, whereas several established reproductive and endocrine risk factors involve exposure over many years. Pregnancy and breastfeeding may also influence long-term breast cancer risk and could offset a small treatment-related effect. These factors may help explain why a clear increase in breast cancer incidence has not been observed in most ART cohorts.

The Postpartum Window: An Under-Recognized Interpretive Lens

Postpartum breast cancer risk is another factor that may complicate interpretation of ART studies. In a pooled analysis of 15 prospective cohorts, Nichols et al. found that breast cancer risk increases transiently after childbirth, with the highest risk occurring approximately five years after delivery before declining over subsequent decades [46]. The increase was greater with older age at first birth and with a family history of breast cancer. Postpartum mammary involution involves inflammatory and stromal changes that may influence the progression of pre-existing lesions [47]. Women who conceive through ART often have their first birth at an older age, and some cohorts have relatively short follow-up periods that overlap with this postpartum risk window. Some of the modest increases reported in parous ART-treated women may therefore reflect the effect of recent childbirth rather than ovarian stimulation itself. Future registry studies should account for time since delivery when evaluating breast cancer outcomes after ART.

Tumor Biology and Molecular Subtype

If ART-related hormonal exposure has a clinically important effect on breast carcinogenesis, an association might be expected to be stronger for hormone receptor-positive tumors. However, most epidemiological studies have reported breast cancer as a single outcome and have not provided sufficient information on estrogen receptor, progesterone receptor, HER2, or intrinsic molecular subtype. The association with carcinoma in situ reported by Williams et al. [31] may also be influenced by differences in detection because in situ lesions are particularly sensitive to screening. Subtype-specific outcome reporting in future studies would help determine whether any small effect is restricted to hormone receptor-positive disease.

Comparison With Previously Published Systematic Reviews and Meta-Analyses

The conclusions of the present synthesis align closely with the quantitative literature accumulated over the past 15 years. In an early meta-analysis, Zreik et al. pooled the then-available studies of fertility drugs and found no overall elevation in breast cancer risk [41]. Li et al. subsequently reported a pooled estimate compatible with a protective effect of IVF in certain subgroups (RR = 0.79, 95% CI 0.65-0.95) [48], while Sergentanis et al., in a systematic review and meta-analysis dedicated specifically to IVF and breast cancer, found no significant association whether the comparator was the general population (RR = 0.91, 95% CI 0.74-1.11) or untreated infertile women (RR = 1.02, 95% CI 0.88-1.18), noting only a marginal, non-significant adverse trend among women younger than 30 years at first treatment [49]. Gennari et al. pooled 20 population-based studies and likewise found no overall excess (summary RR = 1.05, 95% CI 0.96-1.14), although their subgroup analysis suggested a possible increase in non-IVF hormonal treatments followed for more than 10 years [50]. More recently, Barcroft et al. concluded that fertility treatment does not significantly increase the incidence of breast, ovarian, or endometrial cancer [51]; Beebeejaun et al. calculated a summary OR of 0.97 (95% CI 0.90-1.04) with no signal in any drug-specific or cycle-number subgroup [37]; and Cullinane et al. arrived at an essentially identical estimate (OR = 0.97, 95% CI 0.90-1.04), again with neutral subanalyses for CC and other agents [38]. The narrative review of Momenimovahed et al., which surveyed fertility drugs across multiple malignancies, reached the same verdict for breast cancer, observing that the mechanistic plausibility of harm has simply not been borne out by the epidemiological record [52], and the most recent comprehensive appraisal of ART in relation to breast and gynecologic malignancies reiterates this position [53]. The concordance between the present qualitative synthesis and this body of pooled evidence - derived from partially overlapping but differently selected study sets - strengthens confidence in the overall conclusion of no demonstrable association. Where the present review adds to these prior syntheses is in its integration of the adjacent evidence domains discussed above - letrozole pharmacovigilance, postpartum risk dynamics, subtype-specific ascertainment, and the fertility preservation experience - none of which has featured systematically in the earlier reviews of this question.

Women With BRCA1/2 Pathogenic Variants

Women carrying pathogenic BRCA1 or BRCA2 variants are an important subgroup because their baseline breast cancer risk is already elevated and these variants may also be associated with diminished ovarian reserve [54,55]. Three included studies specifically evaluated ART or ovarian stimulation in BRCA carriers and did not demonstrate an increased breast cancer risk associated with treatment [30,34,36]. A systematic review and meta-analysis of women with a family history or BRCA mutations similarly found no significant treatment-associated excess [56]. The number of confirmed carriers exposed to ART in the primary studies remains relatively small, so larger carrier-specific studies are still needed.

Insights From Fertility Preservation in Women With Breast Cancer

Additional evidence comes from women with breast cancer who undergo ovarian stimulation for fertility preservation before gonadotoxic treatment. In this setting, letrozole-based stimulation was developed to limit peak estradiol exposure. Prospective studies found comparable oncological outcomes between stimulated and unstimulated groups [39], and longer follow-up did not demonstrate an excess of recurrence or mortality attributable to stimulation [40]. A systematic review also reported reassuring short- and medium-term outcomes [57]. In addition, pregnancy after breast cancer has not been shown to worsen maternal prognosis, including among women with estrogen receptor-positive disease [58]. These findings provide indirect reassurance about the safety of ovarian stimulation, although they cannot substitute for long-term incidence studies in women without previous breast cancer.

From Historical to Contemporary Stimulation Practice

The epidemiological evidence also reflects major changes in stimulation practice over the period covered by the included cohorts. Much of the literature comes from women treated with older clomiphene regimens, urinary gonadotropins, and long GnRH agonist protocols. Current practice increasingly uses GnRH antagonist protocols, agonist triggering, freeze-all strategies when appropriate, more individualized stimulation, limits on cumulative CC exposure, and letrozole for ovulation induction [4,5]. These differences limit direct extrapolation from historical cohorts to current treatment. The absence of an observed excess risk with older protocols is reassuring, but it does not establish the long-term safety of every contemporary regimen. Long-term follow-up of women treated with current protocols remains necessary.

Clinical Implications: A Proposed Risk-Stratified Counseling Framework

For clinical practice, the available evidence supports counseling women that ART has not been shown to increase breast cancer risk. Women should continue routine, age-appropriate breast screening, and ART exposure alone does not provide an indication for additional surveillance. Women with a strong family history or known BRCA1/2 pathogenic variants should follow established high-risk screening recommendations irrespective of fertility treatment. For women considering numerous stimulation cycles or with substantial historical CC exposure, the available subgroup signals can be discussed as areas of uncertainty rather than as established treatment-related risks. Table 3 provides a proposed framework for counseling and shared decision-making; it is a synthesis of the reviewed evidence rather than a formal clinical guideline.

**Table 3:** Proposed risk-stratified counseling and surveillance framework for breast cancer risk discussions in women undergoing or considering fertility treatment. Abbreviations: ART = assisted reproduction technology; BRCA1/2 = breast cancer susceptibility genes 1 and 2; CC = clomiphene citrate; MRI = magnetic resonance imaging. The framework is a synthesis-derived aid to shared decision-making and should be adapted to national screening policies and individual risk assessment.

Clinical scenario	Summary of the evidence	Suggested counseling and surveillance
General population of women with infertility undergoing ART	Sixteen of 31 primary studies and all major meta-analyses show no significant association with breast cancer incidence [6,8,12,14-16,18,20-22,26,27,30,32,33,36-38,41,48-53].	Reassure that ART has not been shown to increase breast cancer risk; continue standard age-appropriate screening; no additional surveillance on the grounds of ART exposure alone.
High cumulative cycle number (more than four to six stimulation cycles)	Early signals of elevated risk [7,13] not confirmed in the largest cohort [27] or in cycle-stratified meta-analysis [37]; residual confounding likely.	Acknowledge the historical signal and its non-replication; no formal surveillance change; reasonable to discuss cumulative treatment burden when planning repeated cycles.
Prolonged or repeated clomiphene citrate exposure	Excess risk reported in three studies with long follow-up and parity [9,10,28]; not confirmed at the meta-analytic level [37,38]; confounding by indication plausible.	Reassure on current evidence; respect modern cumulative-dose limits; maintain routine screening with a low threshold for clinical breast assessment in long-term historical users.
Letrozole-based ovulation induction	First-line for anovulatory infertility [4,5]; transiently suppresses estradiol; long-term incidence data in healthy infertile women are absent, but safety data from breast cancer survivors are reassuring [39,40].	Explain the favorable theoretical hormonal profile and the current absence of long-term incidence data; routine screening; encourage participation in registry follow-up where available.
BRCA1/2 pathogenic variant carriers	Three carrier-specific studies show no treatment-associated excess [30,34,36], supported by meta-analysis [56]; absolute carrier numbers remain small.	Manage within established high-risk surveillance pathways (annual MRI plus mammography per local protocol) irrespective of ART; ART itself does not modify carrier surveillance; discuss preimplantation genetic testing where appropriate.
Age 40 years or older at treatment initiation	Discordant findings: modest excess in one national cohort [35] versus no excess in a dedicated cohort of women treated at 40 years or older [33].	Counsel on age itself as the dominant risk factor; ensure the patient is enrolled in mammographic screening appropriate for age before commencing treatment.
First five to ten years after an ART-conceived birth	Recent childbirth transiently elevates breast cancer risk in all women, peaking approximately five years postpartum [46]; ART populations concentrate late first births, and postpartum involution biology may promote progression of subclinical lesions [47].	Maintain clinical vigilance for breast symptoms in the early postpartum decade, particularly after a first birth at 35 years or older; investigate persistent breast changes promptly rather than attributing them to lactation.
Breast cancer survivors seeking fertility preservation or pregnancy	Letrozole-supplemented stimulation shows no excess recurrence in controlled series with long-term follow-up [39,40]; systematic review supports short-term safety [57]; pregnancy after breast cancer does not worsen prognosis [58].	Coordinate care with the treating oncologist; letrozole-supplemented protocols preferred in estrogen receptor-positive disease; do not deny fertility preservation on oncological grounds alone.

Strengths and Limitations

The main strengths of this review are the structured multi-database search, explicit eligibility criteria, duplicate screening and data extraction, large aggregate population, long follow-up in several cohorts, and side-by-side presentation of the primary studies. The review also considers additional evidence concerning letrozole, postpartum risk, tumor subtype, and fertility preservation, which helps place the primary epidemiological findings in context. Several limitations must equally be acknowledged. First, this is a narrative review without quantitative pooling; although this design was chosen deliberately in view of the heterogeneity of effect measures and comparators, it precludes a formal summary estimate and formal assessment of publication bias, and no structured risk-of-bias grading of individual studies was undertaken. Second, the included studies were markedly heterogeneous in design, inclusion criteria, comparator selection, confounder adjustment, and outcome expression (OR, RR, HR, and SIR), which complicates direct comparison. Third, confounding by indication is inherent to this field: infertility itself, along with the nulliparity and older age that frequently accompany it, independently influences breast cancer risk, and no observational adjustment can fully separate the treatment from the condition that prompted it. Fourth, surveillance bias may inflate apparent incidence among treated women, who typically maintain closer contact with healthcare systems. Fifth, and perhaps most importantly, much of the evidence derives from women treated with older regimens - some recruited as early as the 1960s - and therefore reflects stimulation practices, drug formulations, and cumulative dose ceilings that differ considerably from the GnRH antagonist protocols, recombinant preparations, agonist trigger strategies, and letrozole-based ovulation induction of the contemporary era; the safety profile of current practice is consequently extrapolated rather than directly measured. Sixth, the contextual literature incorporated in the discussion - on letrozole, postpartum risk, and fertility preservation - was identified through targeted rather than exhaustive searching and was not subject to formal data extraction, and the interpretive framework proposed in Table 3 is a synthesis-derived construct that has not been prospectively validated. Finally, data from several world regions remain sparse, and the carrier subgroup analyses rest on small absolute numbers.

Future Perspectives

Future studies should use prospective registry linkage with standardized definitions of treatment exposure, including drug class, cumulative dose, and number of cycles, and should provide follow-up into the postmenopausal years. Breast cancer outcomes should be reported by hormone receptor status and molecular subtype. Analyses should also account for time since delivery so that postpartum risk can be distinguished from any potential treatment effect. Long-term cohorts of women receiving letrozole-based ovulation induction are particularly needed, as are cohorts treated exclusively with contemporary antagonist and freeze-all protocols. Larger studies of BRCA1/2 carriers would allow more precise estimates for this clinically important subgroup. Harmonization of outcome definitions and effect measures across registries would also facilitate future meta-analyses. Finally, the proposed counseling framework should be evaluated prospectively to determine whether it improves shared decision-making in fertility care.

## Conclusions

Breast cancer is hormonally responsive, and the temporary increase in estrogen and progesterone associated with COS provides a plausible biological basis for concern. However, the epidemiological evidence is generally reassuring. Across 31 primary studies with a cumulative reported sample exceeding 7.1 million participants, and across the major meta-analyses cited in this review, ART has not been convincingly associated with increased breast cancer incidence. Findings concerning multiple treatment cycles, CC, progesterone, and age at treatment initiation have been inconsistent and have not established a clear causal relationship. Evidence from fertility preservation in women with breast cancer is also reassuring, although it addresses recurrence rather than primary cancer incidence. The interpretation of existing cohorts is further complicated by postpartum breast cancer risk, differences in reproductive history, and the fact that many studies evaluated treatment protocols that are no longer standard. Long-term incidence data for letrozole and other contemporary regimens remain limited. Prospective studies with extended follow-up, subtype-specific outcome ascertainment, and appropriate adjustment for time since delivery are therefore needed, particularly for BRCA1/2 carriers and other high-risk groups. In the meantime, the available evidence supports routine breast cancer screening and individualized counseling rather than additional surveillance solely because of ART exposure.
